# The effect of co-dependent (thinking in motion [TIM]) versus single-modality (CogniFit) interventions on cognition and gait among community-dwelling older adults with cognitive impairment: a randomized controlled study

**DOI:** 10.1186/s12877-022-03403-x

**Published:** 2022-08-31

**Authors:** Shiri Embon-Magal, Tal Krasovsky, Israel Doron, Kfir Asraf, Iris Haimov, Efrat Gil, Maayan Agmon

**Affiliations:** 1 Department of Gerontology, Faculty of Social Science and Welfare, University of Haifa, Neve-Yamin, Israel; 2 School of Physical Therapy, Faculty of Social Science and Welfare, University of Haifa, Neve-Yamin, Israel; 3 Department of Gerontology, CRSA – Center for Research & Study of Aging, Faculty of Social Science and Welfare, University of Haifa, Neve-Yamin, Israel; 4 Department of Psychology, University of Haifa, Neve-Yamin, Israel; 5 Department of Psychology, The Max Stern Yezreel Valley College, Neve-Yamin, Israel; 6 Clalit Health Services, Neve-Yamin, Israel; 7 Faculty of Medicine, Technion, Neve-Yamin, Israel; 8 School of Nursing, Faculty of Social Science and Welfare, University of Haifa, Neve-Yamin, Israel

**Keywords:** Brain aging, Motor-cognitive intervention, Remedial intervention

## Abstract

**Background:**

Cognition and motor skills are interrelated throughout the aging process and often show simultaneous deterioration among older adults with cognitive impairment. Co-dependent training has the potential to ameliorate both domains; however, its effect on the gait and cognition of older adults with cognitive impairment has yet to be explored. The aim of this study is to compare the effects of the well-established single-modality cognitive computerized training program, CogniFit, with “Thinking in Motion (TIM),” a co-dependent group intervention, among community-dwelling older adults with cognitive impairment.

**Methods:**

Employing a single-blind randomized control trial design, 47 community-dwelling older adults with cognitive impairment were randomly assigned to 8 weeks of thrice-weekly trainings of TIM or CogniFit. Pre- and post-intervention assessments included cognitive performance, evaluated by a CogniFit battery, as a primary outcome; and gait, under single- and dual-task conditions, as a secondary outcome.

**Results:**

CogniFit total Z scores significantly improved from baseline to post-intervention for both groups. There was a significant main effect for time [F (1, 44) = 17.43, *p* < .001, *η*_*p*_^*2*^ = .283] but not for group [F (1, 44) = 0.001, *p* = .970]. No time X group interaction [F (1, 44) = 1.29, *p* = .261] was found. No changes in gait performance under single and dual-task performance were observed in both groups.

**Conclusions:**

The findings show that single-modality (CogniFit) and co-dependent (TIM) trainings improve cognition but not gait in older adults with cognitive impairment. Such investigations should be extended to include various populations and a broader set of outcome measurements.

**Trial registration:**

ACTRN12616001543471. Date: 08/11/2016.

## Literature review

The older adult population is expected to grow 6–7% per year between 2025 and 2029, resulting in a quadrupling in this century [1]. The presence of dementia in people aged 45 and over in Israel in 2016 was 2.5% of the total population. For those aged 65 and over, the presence was 6.4% and for those aged 85 and over – 22% [2]. Cognitive impairment is associated with an increased risk of disability, higher risk of dementia [3], reduced gait quality and increased risk of falls [3–8]. The relationship between cognition and gait quality is so strong that baseline gait speed is used to predict changes in cognition such as processing speed, memory and executive function [9]. Owing to the interplay between motor and cognitive aging, both cognitive and motor interventions have reciprocal positive effects among older adults. Such interventions can be classified as single-modality (motor or cognitive) or combined training (motor and cognitive) [6, 8]. Indeed, single-modality cognitive intervention shows transfer effects to motor abilities [5] and vice versa [10]. However, the multi-sensory simultaneous reinforcement of the combined multi-modality training may have a greater effect, especially since cognition, motor skills, sensory and temporal processing share neural substrates and functional processes [11]. In addition, older adults appear to use their brains more integrally than young people, as suggested by Harold’s theory [12], and it is possible that this brain behavior present subsequent compensation mechanisms to aid a poor one-modal processing. Multisensory stimulation could, therefore, enhance multi-modal cognitive processing, and especially contribute to low-functioning older adults who suffer from multi-modal impairment, enabling them to rely on more than one domain [9]. The combined multi-modality motor-cognitive interventions can be divided into three types based on the relationships between tasks: (1) independent performance of serial motor and cognitive tasks (either in the same session or in separate sessions), a sequential training shown to be effective mainly when an aerobic motor part precedes the cognitive part of the training [13]; (2) simultaneous performance of tasks i.e., Dual-Task (DT), aiming to improve mainly divided attention [4], found to be effective in improving various motor (e.g., balance and postural control) and cognitive functions (e.g., executive function, memory, and attention) during single and DT conditions [14] (however, a systematic review argues that this training has limited transferability [13]); and (3) simultaneous performance of co-dependent tasks, i.e., an integration of cognitive and motor demands embedded within one task (which cannot be executed separately as in DT), like walking while navigating [15].

Co-dependent tasks represent the requirements of many daily life tasks and are more ecological than other combined trainings such as DT [15]. Although understudied compared to the other two types, co-dependent interventions showed improvement in global cognition [16], executive control, processing speed [17], and balance and motor performance [18–21] among community-dwelling older adults. Evidence also suggests that there is a reduction of the risk of falls following co-dependent training. It seems that the approach of motor and cognitive stimulation allows these two domains to be strengthened not only separately but also as a uniform execution unit [21]. Nevertheless, the potential contribution of co-dependent training to older adults with cognitive impairment is yet to be determined [22].

According to the patient-centered care approach, matching between interventions and specific patients’ needs can optimize the interventions’ effects [23]. Moreover, a simultaneous reinforcement of multisensory systems, as expressed in the co-dependent training, can lead to a greater effect on various systems compared to a single modality training, especially among older adults [9]. To address this question among community-dwelling older adults with cognitive impairment, we developed a co-dependent group training with high adaptability to a variety of cognitive levels called “Thinking in Motion – TIM” [24], and compared its effect on cognition and gait performance to the well-established cognitive computerized training, i.e., CogniFit, among community-dwelling older adults aged 65 and above.

Our primary hypothesis was that the TIM intervention will be as effective as CogniFit in improving global cognition, and more specifically in domains such as working memory, divided attention, processing speed, and visual scanning. These domains were intensively trained in TIM intervention and were shown to be improved after CogniFit training [25]. In addition, considering the specificity of the training of gross motor skills in the TIM intervention, our secondary hypothesis was that TIM will have a higher effect on gait speed and variability during single task (ST) and DT compared to CogniFit.

## Methods

### Eligibility and study design

A single-blind, randomized, and controlled clinical trial compared the effects of TIM, a co-dependent motor-cognitive intervention, and CogniFit, a single modality computerized cognitive training (control), on cognition and gait quality in 47 community-dwelling older adults who participate on a daily basis in leisure activities at the adult day center. Sample size was based on G power calculation with the following parameters: Two groups, measured at two time points; effect size used was the mean Cohen’s *d* of the group differences post intervention of the 16 CogniFit measures in Haimov & Shatil (2013), which was 0.635 (Cohen’s ƒ = 0.318); α was 0.05; power to detect an effect was 95%; correlations between time points were assumed to be 0.2. Potential participants were recruited through referrals from day center staff members. Inclusion criteria were: (1) age over 65; (2) ability to walk independently with or without an assistive device; (3) ability to understand simple instructions and sign an informed consent form; (4) ability to commit to the program based on a short interview. This set of minimum criteria seeks to represent as reliably as possible the population of the day centers. Subjects with a MoCA [26] score lower than 18 underwent a follow-up interview to examine their eligibility to participate in the study. Participants who met the inclusion criteria were randomly assigned to an eight-week, thrice per week, 40-minute intervention in one of two groups: a TIM group intervention (*n* = 28), which was split into smaller training groups that contained 14 practitioners in each group; or an individual computerized cognitive training group using CogniFit training (*n* = 19) (i.e., control group). Randomization was conducted with a concealed allocation using a table with a random number that was generated by a statistician. Levene’s test of homogeneity of variance was not significant for any of the measures, and Box’s M test of equality of covariance matrices was not significant for any of the repeated measures ANOVA tests. Baseline assessment was conducted by a research assistant, naive to participant assignment, and included: (1) demographic and psychological measures: The Geriatric Depression Scale (GDS) [27], Short Anxiety Screening Test (SAST) [28] and the Activities-specific Balance Confidence (ABC) Scale [29]; (2) cognitive performance measures: Montreal Cognitive Assessment, MoCA [26] and a computerized neuro-cognitive assessment using CogniFit battery [30]; and (3) gait assessment in single and dual-task conditions using an accelerometer and gyroscope mounted on the waist from McRoberts Mobility Lab [31]. Cognitive and gait evaluations were conducted again after 8 weeks of intervention. The study protocol was approved by an institutional review board. All participants signed a consent form and did not receive any monetary compensation for participation.

#### Thinking in motion (TIM) intervention

**Fig. 1:**
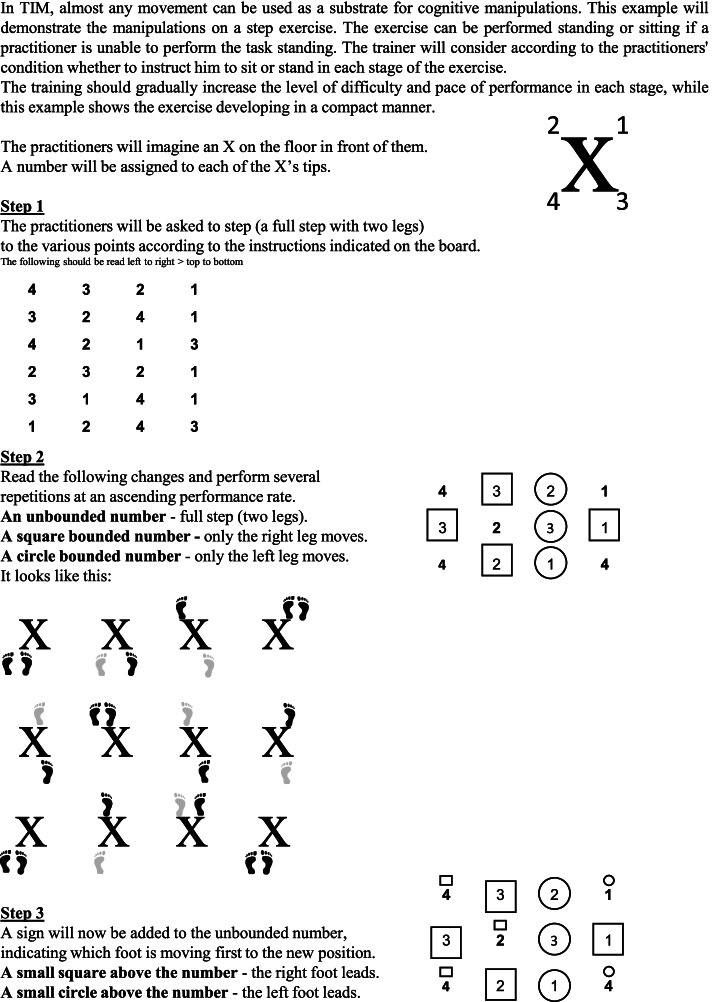
Example of manipulation option in TIM

TIM is a combined motor-cognitive co-dependent intervention inspired by the Eshkol-Wachman Movement Notation (EWMN), which uses graphic symbols to describe motion [32]. Unlike the fixed use of graphic symbols in EWMN, however, TIM departs from the permanent system of symbols and uses the ephemerality of symbols and frequent changes in them as a central tool for cognitive stimulation. The ephemerality and frequent changes require a renewed learning process, coping with coding permanence and mental flexibility. In addition, the use of symbols creates the cognitive challenge of producing movement from interpreting a graphic sign, i.e., visual scanning, information processing, and spatial perception. The symbols represent various components in movement such as organ movement, landmarks in space, time, and rhythm elements. After establishing the given symbols for the exercise, a cognitive challenge is graded by a constant change of the symbols’ order. The TIM trainer uses a “manipulation bank” that is applied to various components of the movement or how they are presented graphically. The chosen manipulations enable the TIM trainer to manage the level of difficulty and the variety of cognitive skills required to perform them. An example of TIM training and manipulation options is shown in Fig. 1.

#### Control group- CogniFit intervention

CogniFit is a computerized cognitive training program which was found to be effective in improving cognition and gait [7, 25]. The program was personally tailored based on a 45-minute baseline assessment [30]. Each training session included a mixture of auditory, visual, and cross-modality tasks aimed at training executive functions, attention, and other cognitive processes. Verbal instructions were written on the screen before each task and then demonstrated by the program [33]. Technical support in operating the software and assistance in understanding the tasks were available.

### Outcome measures assessments

Measures were undertaken at baseline and after the eight-week intervention. All assessments were conducted by a blinded research assistant.

#### Primary outcome: cognitive functions

The 40-minute CogniFit computerized neurocognitive assessment battery [30] was used to evaluate changes in cognitive function following both interventions. Apart from the global score, the cognitive indicators examined in this study were working memory, divided attention, processing speed, and visual scanning. These tests were found to have good internal consistency (Cronbach’s alpha = 0.85–0.88), and test-retest reliability (*r* = 0.69–0.92). The CogniFit assessment battery also has validity, as it is based on well validated cognitive tests [34].

#### Secondary outcome: gait performance

Gait measures included gait speed and stride time variability collected under ST and DT conditions with the McRoberts Mobility Lab, an accelerometer and gyroscope attached to the participants’ waists [35] which measured temporal variables. Gait with DT is a well-established marker to describe progression of dementia and cognitive impairment. The link between cognition and gait control has been sufficiently demonstrated [4]. Gait tasks took place for periods of 1 min along a six-meter route in a quiet room. Assessment started in a static position, and the participants were instructed to walk at their usual pace. One trial for each conditions (single and dual task) was performed. Turn periods were discarded, and gait variability was calculated using the total number of strides by dividing standard deviation for the gait variable by its mean. With according to previous studies [36, 37], the mean and the standard deviation of gait cycles’ number used to measure gait variability was 29.72 ± 9.57 for single task, and 24.89 ± 10.91 for dual task. The cognitive task attached to the walking was subtraction by 3 from a random number between 100 and 250 [38]. The order of the tasks was randomized.

## Analysis

All analyses were performed using SPSS 26 (IBM SPSS Statistics, New York, US). Continuous data are presented as mean and standard deviation or median and interquartile range, while categorical data are presented as frequencies (percentage and number of participants). Between-group differences in demographic data were analyzed via Mann-Whitney U tests, Pearsons’ chi-square test, or Fisher’s Exact tests. Global and domain-specific CogniFit measures, presented as mean ± standard deviation at baseline and post-intervention were normalized (z-scored) to normative data of the population obtained from the CogniFit database, and were examined using between-within repeated measures Analysis of variance (ANOVA). When an interaction was significant, it was followed by t-test post-hoc analyses. Gait outcomes were speed and stride time variability. Effect size estimator was partial eta-squared (*ηp*^*2*^) for the ANOVA test. Effect sizes are reported for significant comparisons only. Data deviating more than |2.5| standard deviation from group mean were considered outliers and were excluded from statistical analysis. Data were analyzed with an intention-to-treat approach.

## Results

### Group characteristics

**Fig. 2:**
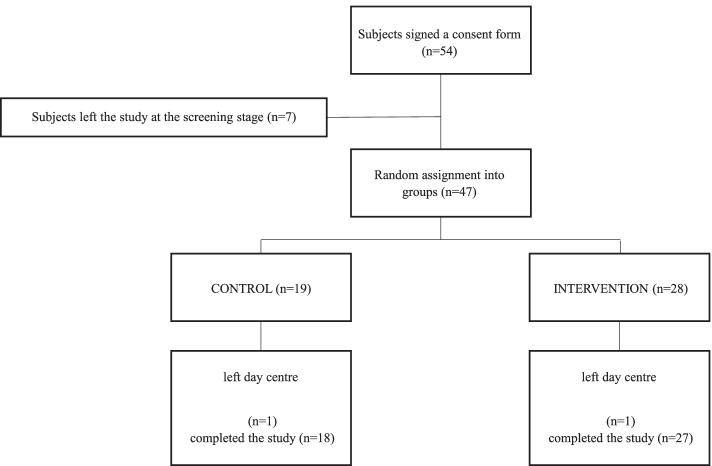
CONSORT Flow of participants

A total of 54 participants were referred to the research team by the center staff. Seven participants withdrew from the study before group assignment: five did not meet the inclusion criteria and two declined to participate (Fig. 2). Thus, the sample was comprised of 30 women and 17 men, with a mean age of 81.16 years (SD = 8.23). No differences between groups in age, years of education, gender, smoking, cardio-metabolic diseases, stroke, balance efficacy, anxiety, depression, and cognitive abilities at baseline were found (Table 1). It is notable that the participants in this sample were frail older adults with low gait speed and MoCA scores that are associated with moderate to severe cognitive impairment. No subjects were diagnosed with dementia; however, as noted above, subjects with a MoCA [26] score lower than 18 underwent a follow-up interview to examine their eligibility to participate in the study.

**Table 1:** Comparisons of demographic measures between the groups at baseline

Parameter	Control (***n*** = 19)	Intervention (***n*** = 27)	***U / t / χ2*** (df)	***P***
Age	Media*n* = 82 IQR = 70–84	Median = 85 IQR = 77–88	148	.197
Years of Education	Media*n* = 10 IQR = 7–13	Median = 8.5 IQR = 8–12	171.5	.664
% of Females	68.42 (*n* = 13)	62.96 (*n* = 17)	0.29 (1)	.758
% of No Smoking	100 (*n* = 19)	100 (*n* = 27)	–	–
% of Falling in the Last Year	42.10 (*n* = 8)	51.85 (*n* = 14)	0.42 (1)	.514
% of Cardio Metabolic Disease	52.63 (*n* = 10)	77.77 (*n* = 21)	3.20 (1)	.073
% of Stroke	15.78 (*n* = 3)	25.92 (*n* = 7)	^	.488
Balance Efficacy (ABC- scale 0–100)	Median = 58.7 IQR = 40.4–70.9	Median = 60 IQR = 34.3–80	188.5	.855
Anxiety (SAST- scale 10–40)	Media*n* = 31.5 IQR = 27.2–33	Median = 29 IQR = 26–32	135.5	.167
Depression (GDS - scale 0–30)	Median = 2 IQR = 1–4.5	Median = 4 IQR = 1–6.2	182	.397
Cognition (MoCA- scale 0–30)	Median = 16 IQR = 12–23	Median = 16 IQR = 11–19	229.5	.553
CogniFit global cognition	Mean = − 3.82 SD = 1.78	Mean = − 3.96 SD = 1.20	0.32 (44)	.747
Gait Speed at Baseline (m/s)	Mean = 0.59 SD = 0.34	Mean = 0.47 SD = 0.21	1.56 (43)	.125
Walking aids (%)	52.6	53.6		0.94

^ Based on Fisher’s Exact Test. Cardio metabolic disease defined as at least one of the following diagnoses: high blood pressure, heart condition or diabetes. *Abbreviations*: *ABC* Activities-specific Balance Confidence (higher score denotes higher confidence),  *SAST* Short Anxiety Screening Test (higher score denotes higher level of anxiety),  *GDS* Geriatric Depression Scale (higher score denotes higher level of anxiety),  *MoCA* Montreal Cognitive Assessment (higher score denotes higher cognitive performance)

The means and standard deviations of the groups in the study’s outcome measures are presented in Table 2.

**Table 2:** Means and standard deviations of the study’s outcome measures

Parameter	Baseline		Post intervention	
*Primary Outcomes*	**Control**	**Intervention**	**Control**	**Intervention**
Global Cognition	Mean = − 3.82 SD = 1.78	Mean = − 3.96 SD = 1.20	Mean = − 3.39 SD = 1.43	Mean = − 3.22 SD = 1.35
Working Memory	Mean = − 3.86 SD = 1.96	Mean = − 4.14 SD = 1.31	Mean = − 3.35 SD = 1.45	Mean = − 3.45 SD = 1.26
Divided Attention	Mean = − 2.30 SD = 0.40	Mean = − 2.26 SD = 0.55	Mean = − 1.88 SD = 0.72	Mean = − 2.23 SD = 0.50
Processing Speed	Mean = − 4.90 SD = 2.96	Mean = − 4.07 SD = 1.71	Mean = − 4.31 SD = 2.42	Mean = − 3.34 SD = 2.07
Visual Scanning	Mean = − 2.32 SD = 2.12	Mean = − 2.25 SD = 1.77	Mean = − 2.96 SD = 2.17	Mean = − 1.63 SD = 2.03
*Secondary Outcomes*	**Control**	**Intervention**	**Control**	**Intervention**
Gait Speed (ST)	Mean = 0.59 SD = 0.34	Mean = 0.47 SD = 0.21	Mean = 0.56 SD = 0.32	Mean = 0.50 SD = 0.17
Gait Speed (DT)	Mean = 0.42 SD = 0.21	Mean = 0.31 SD = 0.13	Mean = 0.41 SD = 0.18	Mean = 0.30 SD = 0.12
Stride Time Variability (ST)	Mean = 0.18 SD = 0.17	Mean = 0.12 SD = 0.09	Mean = 0.15 SD = 0.15	Mean = 0.12 SD = 0.09
Stride Time Variability (DT)	Mean = 0.18 SD = 0.11	Mean = 0.19 SD = 0.11	Mean = 0.17 SD = 0.09	Mean = 0.22 SD = 0.15

### Primary outcomes

**Fig. 3:**
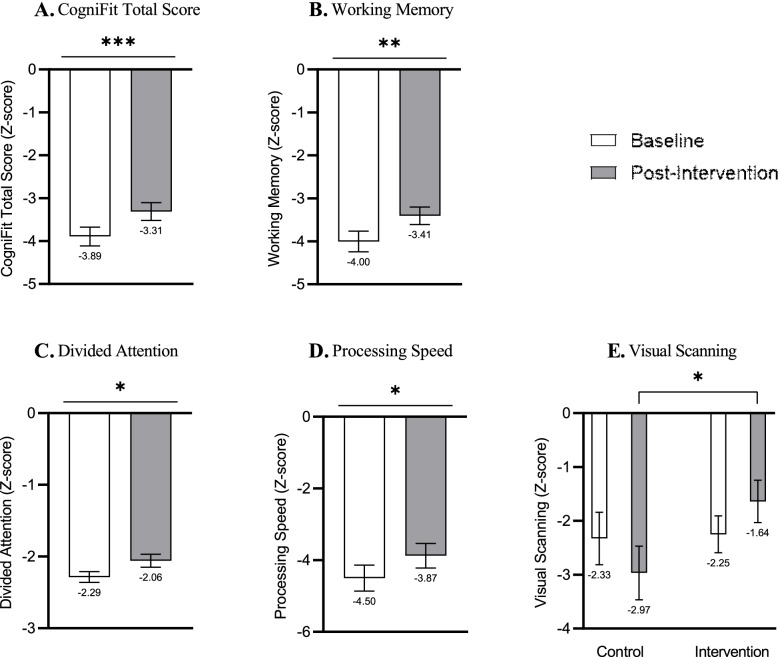
Effect of interventions on global cognition and on specific domains. *p < .05, **p < .01, ***p < .001.

Results are presented in Fig. 3. Compared with baseline, both groups improved in cognition as measured by the CogniFit test. Analysis of CogniFit total score (Z-scored) revealed a significant main effect for time [F (1, 44) = 17.43, *p* < .001, *η*_*p*_^*2*^ = .283] such that the cognitive performance at post-intervention (M = -3.31, SD = 1.40) improved significantly in both groups, compared to the cognitive performance at baseline (M = -3.89, SD = 1.49), with a large effect size. No main effect for group [F (1, 44) = 0.001, *p* = .970], or a time X group interaction [F (1, 44) = 1.29, *p* = .261] were found. Similar results were demonstrated in specific cognitive domains: working memory following intervention (M = -3.4, SD = 1.36) improved compared to baseline (M = -4.00, SD = 1.63), with a large effect size [F (1, 44) = 10.97, *p* = .001, *η*_*p*_^*2*^ = .199], with no main effect for group or a time X group interaction. Divided attention improved following intervention with a medium effect size [F (1, 44) = 5.54, *p* = .023, *η*_*p*_^*2*^ = .111] from (M = -2.28, SD = 0.50) to (M = -2.05, SD = 0.61). No main effect for group was found, but the time X group interaction [F (1, 44) = 3.90, *p* = .054] was closer to being statistically significant. Processing speed post-intervention (M = -3.87, SD = 2.24) improved compared with baseline (M = -4.50, SD = 2.38) with a medium effect size [F (1, 41) = 5.73, *p* = .021, *η*_*p*_^*2*^ = .122], and no effect for group or time X group interaction were found. In contrast, in the visual scanning domain, a significant time X group interaction emerged [F (1, 44) = 4.63, *p* = .036, *η*_*p*_^*2*^ = .095] with a medium effect size. Post-hoc analysis revealed that while there was no difference between the groups at baseline [t (44)= − 0.13, *p* = .893], there was a significant difference at post-intervention [t (44)= − 2.11, *p* = .039], so the TIM group (M = -1.63, SD = 2.03) improved more than CogniFit (M = -2.96, SD = 2.17). Paired-samples t-tests showed no difference within the groups (not shown).

### Secondary outcomes

Analyses of gait speed under ST condition revealed no main effect for time [F (1, 42) = 0.02, *p* = .876] or group [F (1, 42) = 1.60, *p* = .212], and no significant time X group interaction [F (1, 42) = 2.23, *p* = .142]. On the other hand, under DT condition, a main effect for group was found [F (1, 43) = 5.36, *p* = .025, *η*_*p*_^*2*^ = 0.11], so the control group had a higher mean gait speed (M = 0.41, SD = 0.15) compared to the intervention group (M = 0.31 m / seconds, SD = 0.15) at both time points. No main effect for time [F (1, 43) = 0.05, *p* = .816] or time X group interaction [F (1, 43) = 0.02, *p* = .869] were found. Variability of stride time under ST did not differ between or within the groups, so no effects for time [F (1, 38) = 0.22, *p* = .635], group [F (1, 38) = 1.34, *p* = .253], or interaction between them [F (1, 38) = 0.005, *p* = .942] were found. Variability of stride time under DT also did not differ between or within the groups, so no effects for time [F (1, 37) = 0.35, *p* = .555], group [F (1, 37) = 1.06, *p* = .307], or interaction between them [F (1, 37) = 0.63, *p* = .429] were found.

## Discussion

The findings of this single-blind randomized clinical trial demonstrate cognitive improvement in both interventions, i.e., TIM and CogniFit, among community-dwelling older adults with cognitive impairment. The intervention group average performance was 82.5% of all the sessions that took place. In the control group, 90% of the participants received assistance in connection with the training software program. However, their adherence was low, with completion rate at around 30%. Most of our findings support the first hypothesis, suggesting similar global cognitive improvement, as well as similar improvement in specific domains, i.e., working memory, divided attention, and processing speed among both groups. These findings are in line with previous findings [16, 17, 20, 25, 39]. One exception is the visual scanning domain, which, in contrast to our hypothesis, was improved only among TIM participants. Indeed, visual scanning is a central requirement within TIM training due to its extensive use of graphical tools. Unlike our findings, a previous study by Shatil showed improvement in visual scanning following 8 weeks of CogniFit training among community-dwelling older adults [25]. This discrepancy can be explained by the different cognitive abilities of the participants in the current study and Shatil’s research [25], probably thus limiting the participants in the control group (CogniFit) from benefiting from its advantages. Notwithstanding, such cognitive results are encouraging, especially given the participants’ relatively low baseline cognitive scores [40], and further support the potential for cognitive improvement even among older adults with cognitive impairments [41]. Such potential is supported by the remedial model that stresses the ability of training to reinforce brain plasticity [42, 43].

Our second hypothesis, that both groups would show motor improvement with an advantage for the TIM group, was not supported and no significant improvement in gait performance was observed among participants in either group. These findings are inconsistent with a previous study that demonstrated gait improvement after thrice-weekly, eight-week CogniFit training among high-functioning older adults [7] and with studies that evaluated the effect of various co-dependent trainings on various older adult populations [18, 20, 39, 44, 45]. The difference between findings may stem from the differences in baseline cognitive statuses between the participants in the above-mentioned studies. The learning abilities of those with cognitive impairment are limited [41]. In the current study, due to safety reasons the intervention was conducted while sitting, similar to the control group, yet both groups maintained their gait abilities during the intervention period. Indeed, maintenance of abilities is often the main goal with older adults with cognitive impairment [46].

This study contained several limitations. The first is that although the composition of the population in the study faithfully represents the composition of the population that reaches the day centers, this also has research limitations. The sample is homogenous in several aspects such as socio-economic status and general functioning, but the cognitive score is widely varied. Indeed, the objective cognitive score is associated with various factors. While this wide range of cognitive function may represent the general population, it can confound the findings and limit their generalizability to different populations such as higher functioning older adults or patients with neurological diseases. Second, due to the small sample, we did not control for gender in the analysis. Third, the cognitive assessment was conducted using the CogniFit assessment tool which was similar to the training of CogniFit. Yet, the fact that both groups similarly improved in this test emphasizes the transferability of TIM training. Fourth, the motor effects of the interventions were examined only on gait in a laboratory setting, limiting their transferability to other daily functions. Fifth, it is hard to infer a causal pathway between the interventions and the preserved gait performance, due to the lack of a waiting list control group. Sixth, due to the limited number of participants and limited resources, unbalanced randomization was conducted and the intervention group was larger than the control group; however, given that intervention effectiveness for this group has been examined in previous studies, correction was not made. Seventh, the effect of the difference between settings (individual vs. group) could not be controlled and the adherence rate was higher in the intervention group. Future studies should be conducted among various populations that explore broader motor and social abilities (e.g., participation) in ecological settings and incorporate a waiting list control arm.

## Conclusions

Our findings demonstrate that among community-dwelling older adults with cognitive impairment, 8 weeks of thrice-weekly interventions (TIM and CogniFit) may contribute to global cognition, working memory, divided attention, and processing speed. TIM also demonstrated an improvement in visual scanning. No change in gait performance was observed among participants in both groups. Older adults can benefit from the advantages of both interventions, supporting personalization of treatment plans. Future studies should evaluate TIM among a broader population of older adults in different settings to further address the effect of co-dependent combined training compared to a single modality intervention on motor abilities and cognition throughout the aging process.

## Data Availability

The datasets used and/or analyzed during the current study are available from the corresponding author on reasonable request. The study is registered in ANZCTR, under registration number ACTRN12616001543471, where the full protocol is available.

## Abbreviations

TIM: Thinking In Motion
DT: Dual Task
ST: Single Task
